# Identification of pathogen(s) in infectious diseases using shotgun metagenomic sequencing and conventional culture: a comparative study

**DOI:** 10.7717/peerj.11699

**Published:** 2021-06-29

**Authors:** Huan Chen, Jun Li, Shanshan Yan, Hui Sun, Chuyi Tan, Meidong Liu, Ke Liu, Huali Zhang, Mingxiang Zou, Xianzhong Xiao

**Affiliations:** 1 Postdoctoral Research Station of Clinical Medicine & Department of Hematology, Third Xiangya Hospital, Central South University, Changsha, China; 2 Department of Clinical Laboratory, Xiangya Hospital, Central South University, Changsha, China; 3 Department of Intensive Medicine, Third Xiangya Hospital, Central South University, Changsha, China; 4 Sepsis Translational Medicine Key Lab of Hunan Province, Department of Pathophysiology, School of Basic Medicine Science, Central South University, Changsha, China

**Keywords:** Shotgun metagenomic sequencing, Culture, Pathogen identification, Infectious disease, Antibiotic resistance

## Abstract

**Background:**

Early and accurate diagnosis of microorganism(s) is important to optimize antimicrobial therapy. Shotgun metagenomic sequencing technology, an unbiased and comprehensive method for pathogen identification, seems to potentially assist or even replace conventional microbiological methodology in the diagnosis of infectious diseases. However, evidence in clinical application of this platform is relatively limited.

**Methods:**

To evaluate the capability of shotgun metagenomic sequencing technology in clinical practice, both shotgun metagenomic sequencing and conventional culture were performed in the PCR-positive body fluid specimens of 20 patients with suspected infection. The sequenced data were then analyzed for taxonomic identification of microbes and antibiotic resistance gene prediction using bioinformatics pipeline.

**Results:**

Shotgun metagenomic sequencing results showed a concordance of 17/20 compared with culture results in bacterial detection, and a concordance of 20/20 compared with culture results in fungal detection. Besides, drug-resistant types annotated from antibiotic resistance genes showed much similarity with antibiotic classes identified by susceptibility tests, and more than half of the specimens had consistent drug types between shotgun metagenomic sequencing and culture results.

**Conclusions:**

Pathogen identification and antibiotic resistance gene prediction by shotgun metagenomic sequencing identification had the potential to diagnose microorganisms in infectious diseases, and it was especially helpful for multiple microbial co-infections and for the cases where standard culture approached failed to identify microorganisms.

## Introduction

Infectious diseases remain a major causes of death throughout the world (World Health Organization, 2020), and pathogen identification is essential for its diagnosis and treatment (Rhodes et al., 2017). Currently, culture-based approaches are still considered the gold standard for diagnosis of bacterial or fungal infection (Vincent et al., 2015), and culture-associated drug sensitive test provides effective information for antimicrobial therapy. However, some microorganisms may not easy to identify by routinely culture methods used in clinical microbiology department under the influence of many factors, such as non-viable bacteria, fastidious growth requirements, inhibition by other organisms, etc. (Grumaz et al., 2016; Gu et al., 2021). These factors make accurate diagnosis and treatment of infections a challenge, which might lead to unnecessary use of broad-spectrum antibiotics and increase the risk of microbial resistance and expense of hospitalization.

Shotgun metagenomic sequencing approach, as an unbiased, sensitive and high-throughput approach, has become more attractive to clinical microbiology laboratories for its fast detection of possible pathogens directly in a clinical sample without nucleonic acids enrichment or prior region selection and is especially suitable for unknown, atypical, and emerging etiologies of complex infectious diseases (Miao et al., 2018; Thoendel et al., 2018; Zhou et al., 2019). In addition, antibiotic resistance genes (ARGs) prediction by shotgun metagenomic sequencing shows the potential to determine appropriate antimicrobial therapy and guide clinical management (Crofts, Gasparrini & Dantas, 2017; Wang et al., 2017). Furthermore, shotgun metagenomic sequencing seems to be less influenced by prior application of antibiotics (Miao et al., 2018; Zhang et al., 2019). In view of these giant advantages, shotgun metagenomic sequencing technology might probably become a routine method in the near future, partly taking the place of conventional culture (Goldberg et al., 2015). However, literature relevant to clinical applications has mostly focused on its partial function of rapid detection in certain infectious diseases (e.g., central nervous system infections, respiratory infections, bone and joint infections, etc. (Fang et al., 2020; Huang et al., 2020; Wilson et al., 2019; Yan et al., 2020; Zhang et al., 2020)). On the other hand, to comprehensively interpret and evaluate the full-function of shotgun metagenomic sequencing (including pathogen identification and ARGs prediction) remains challenging, which needs more investigation and validation. For these purposes, the present study has been performed to expand shotgun metagenomic sequencing detection in real-world clinical practice while completely estimating its performance in both detecting causative microbes and providing resistance information.

## Materials & methods

### Ethics statement

The Ethics Committee of the Xiangya Hospital of Central South University approved the study (No. 201703567), and written informed consents were obtained from all patients. All procedures were carried out in accordance with the Declaration of Helsinki and all experiments were performed by the relevant guidelines and the institutional regulations.

### Patient recruitment

**Figure 1:**
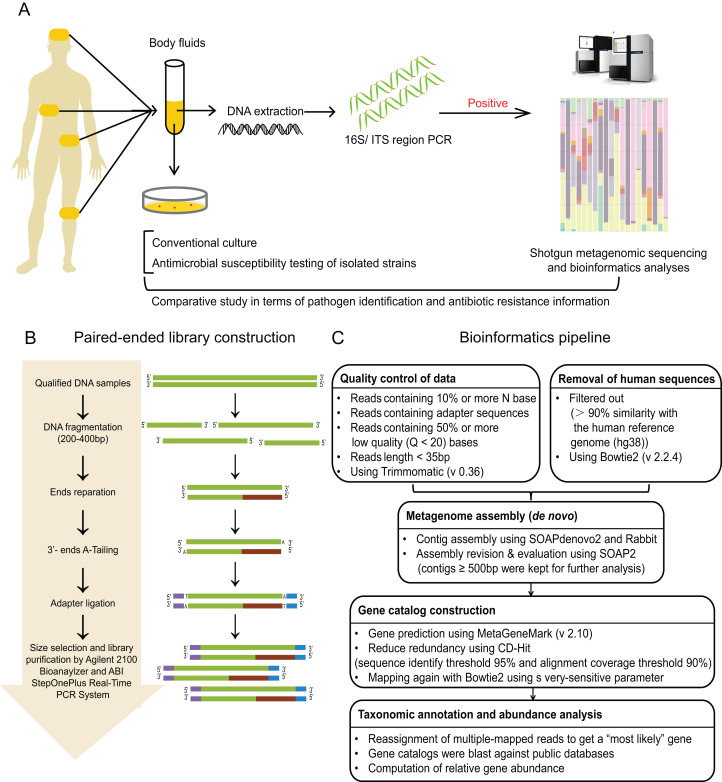
Study workflow. (A) Schematic of comparative study workflow. Patients’ samples of body fluids were collected for conventional culture and 16S/ITS region PCR test. PCR-positive specimens which passed the quality control of sequencing were selected to perform shotgun metagenomic sequencing and bioinformatics analyses, and further comparative study between culture results and sequencing results. (B) Schematic of paired-end library construction in accordance with Illumina’s instruction. (C) Bioinformatics pipeline for shotgun metagenomic sequencing.

**Figure 2:**
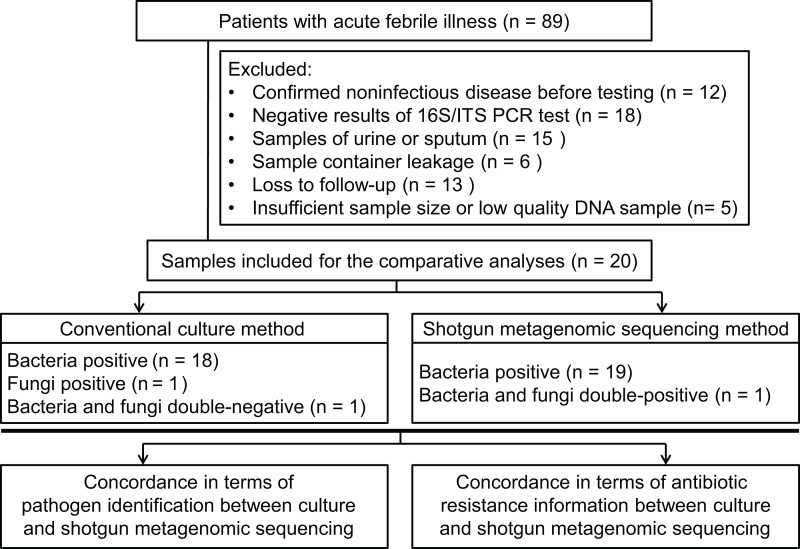
Flowchart for enrollment.

Patients (18 years or older), admitted to hospital, with acute febrile illness were recruited at the Xiangya Hospital in Changsha, China, between March 2017 and May 2017. Patients were considered for inclusion with a suspected diagnosis of bacterial or fungal infection. Infection was defined as an axillary temperature of more than 38 °C or less than 35 °C; increased or decreased white blood cell count (>12 or <4 cells/mL^3^); and/or clinical signs associated with local or systemic infection, such as abscess, respiratory distress, prostration, altered consciousness, convulsions, clinical jaundice, signs of shock, etc.; and/or other infection associated radiographic results. Using our inclusion criteria, 89 patients were included to collect samples to perform bacterial/fungal PCR amplification tests to get candidate samples for the shotgun metagenomic sequencing and routine culture in a pairwise manner. After primarily screening and exclusion, specimens from 20 patients were finally subjected to the follow-up testing and enrolled into the comparative study between shotgun metagenomic sequencing and traditional methodology (see Figs. 1A and 2). Detailed information of participated patients was collected and listed in Table 1.

**Table 1:** Demographics of patients and sample characteristics.

Variables (*n* = 20)	Value
Patient demographics	
Age (years), median (*IQR*)	57 (44–62)
Gender, Male, *n* (%)	12 (60)
Days hospitalized, median (*IQR*)	17 (12–27)
Immunocompromised, *n* (%)	6 (30)
Empirical antibiotics use before sampling, *n* (%)	18 (90)
Temperature (°C), median (*IQR*)	38.5 (38–38.8)
WBC count (10^9^/L), median (*IQR*)	15.4 (12.8–18.4)
Neutrophils (%), median (*IQR*)	89.15 (82.93–94.90)
Procalcitonin (ng/mL), median (*IQR*)	1.87 (0.49–55.92)
SOFA score, median (IQR)	2 (0–5)
Sample features	
Sample type, *n* (%)	
Abscess	5 (25)
Cerebrospinal fluid	5 (25)
Bile	4 (20)
Others*	6 (30)
Positive culture rate, *n* (%)	
Bacteria positive	18 (90)
Fungi positive	1 (5)
Time to final culture result (days), median (*IQR*)	4 (3–4)

**Note:**

* Including ascites, bone marrow, joint aspirate (knee), and pleural fluid.

### Sample collection, culture, and susceptibility testing

Sterile fluid specimens of suspected infection site were collected from the Microbiology department of the Xiangya Hospital within 48 h of infection, including cerebrospinal (CSF), pleural, peritoneal, or synovial fluid. Urine and sputum samples were not considered. The laboratory used standard aerobic culture methods for processing clinical specimens and they conformed to standard protocols published for clinical microbiology laboratories for detection of pathogens and interpretation of results (Jorgensen et al., 2015). Agar plates and BHI broth plates were used to culture samples of sterile sites, and incubated routinely for 5 days to observe bacterial growth. According to the Clinical and Laboratory Standards Institute (CSLI) guidelines (CLSI, 2015), antimicrobial susceptibility testing of isolated strains was carried out by Vitek 32 automated system (bioMérieux, Marcy-l’Étoile, France), and was identified by matrix-assisted laser desorption ionization time-of-flight mass spectrometry (MALDI-TOF MS) (Bruker Daltonik, Gmbh, Berlin, Germany) with standard protocols.

### DNA extraction

The remaining specimens (that had been processed for culture) were stripped of patient identification and coded before experiments. The specimens were transferred to the laboratory for polymerase chain reaction (PCR). Sample DNA extraction was carried out using QIAAmp DNA Mini kit (Qiagen, Hilden, Germany) according to manufacturer’s instructions. The quality of DNA was analyzed using Qubit (Invitrogen, Carlsbad, CA, USA) and checked by 1% agarose gel electrophoresis. Extracted DNA was stored at −80 °C.

### PCR amplification

The presence of bacterial or fungal DNA (or both) was confirmed by using PCR amplification with the common primers of bacterial 16S gene region and fungal ITS1/4 gene region. To control the contamination, extracted DNA was tested in batches with positive and negative controls for bacteria and fungi, respectively. PCR products were run on a 2% agarose gel and visualized using nucleic acid dyestuffs. Strains of *E.coli* (ATCC: 25922) and *C. albicans* (ATCC: 10231) were used as positive controls (Abayasekara et al., 2017). Only specimens with positive PCR results were selected for and sequencing.

PCR amplification was performed as mentioned below: 95 °C for 2 min, 30 cycles at 95 °C for 30 s, 57 °C for 30 s and 72 °C for 60 s, and one cycle at 72 °C for 10 min for 16S amplification; and 94 °C for 5 min, 30 cycles at 94 °C for 30 s, 55 °C for 30 s and 72 °C for 60 s, and one cycle at 72 °C for 7 min for ITS amplification, respectively (Abayasekara et al., 2017). Primers were shown as follows: 16S rDNA F: 5′-ACTCCTACGGGAGGCAGCAGT-3′, R: 5′-TGACGGGCGGTGTGTACAAG-3′; ITS1: 5′-TCCGTAGGTGAACCTGCGG-3′, ITS4:5′-TCCTCCGCTTATTGATATGC-3′ (Sangon Biotech, Shanghai, China).

### Sequencing and bioinformatics analyses

The workflow of sequencing and bioinformatics analyses was performed as follows (see Figs. 2B and 2C), including library construction, sequencing, quality control and host sequence removal, metagenome assembly, gene prediction and taxonomic classification, calculation of relative gene abundance, and prediction of ARGs.

#### Library construction

Library construction was performed using Illumina library preparation kit according to the manufacture’s instruction (Illumina, San Diego, CA, USA) (Liu et al., 2017). To be brief, qulified DNA samples were sheared into small fragments (about 200–400 bp) by Covaris. After ends reparation, 3′-ends-adenylation, and adapter ligation, adequate size of DNA fragments were selected by Ampure beads. Then, Agilent 2100 Bioanaylzer and ABI StepOnePlus Real-Time PCR System were used to qualify and quantify the sample libraries. The qualified libraries were sequenced via Illumina platform (BGI, Shenzhen, China).

#### Sequencing process

All 20 samples were sequenced on Illumina HiSeq 2500 platform (paired end; insert size, 350 bp; read length, 150 bp) via Beijing Genomics Institute in accordance with the manufacturer’s instructions (BGI, Shenzhen, China). To control the contamination, a negative control with only reagents was added in each run. All the raw reads were deposited in the Sequence Read Archive (SRA) of the National Center for Biotechnology Information (http://www.ncbi.nlm.nih.gov/) with accession number PRJNA527188.

#### Quality control and removal of host sequences

In order to avoid contamination and acquire reliable results, unqualified reads and short reads were removed using Trimmomatic (version 0.36) (Bolger, Lohse & Usadel, 2014), including: (i) reads containing 10% or more N base; (ii) reads containing adapter sequences; (iii) reads containing 50% or more low quality (Q < 20) bases; (iv) reads length < 35 bp. Then, the host-related reads were filtered out which mapped to the human reference genome (hg19) with more than 90% similarity using Bowtie2 (version 2.2.4). Thus, high quality reads for these 20 samples were obtained for the next step.

#### De novo metagenome assembly

The filtered reads were assembled de novo with SOAPdenovo2 (Grabherr et al., 2011), and further assembled with Rabbit (You et al., 2013) to obtain longer contigs. These longer reads were mapped back to SOAP2 (Li et al., 2009), and only contigs no less than 500 bp (with the optimal *k-mer*) were kept as clean data depending on both contig *N50* and mapping rate, and applied for further functional analysis.

#### Taxonomic classification

We used MetaGeneMark (version 2.10) (Zhu, Lomsadze & Borodovsky, 2010) for gene prediction, and CD-Hit (Li & Godzik, 2006) for clustering genes in the whole cohort. The high quality genes after filtering were merged to generate gene catalog using Bowtie2 (on average of 10× coverage depth). Then, these selected genes were blasted against public databases, performed by the MEGAN (version 4.6) to classified these genes into four microbial genome databases (bacteria, fungi, viruses, and parasites) using LCA algorithm (Huson et al., 2007). The classification reference databases were downloaded from the National Center for Biotechnology Information (NCBI; https://ncbi.nlm.nih.gov/genomes/). Notably, reads who mapping to multiple genes were reassigned to obtain a most possible gene by Pathoscope (version 1.0) (Francis et al., 2013), which could resubmit the reads to a Bayesian framework to examine the global similarity of sequence.

#### Computation of relative gene abundance

After classification, the relative abundance of any sample *S* was calculated by the formula below (Qin et al., 2012):

$${a_i} = {\rm \; }\displaystyle{{{b_i}} \over {\mathop \sum \nolimits_j {b_j}}} = \; \displaystyle{{\textstyle{{{X_i}} \over {{L_i}}}} \over {\mathop \sum \nolimits_j \textstyle{{{X_j}} \over {{L_j}}}}}$$

where *a*_*i*_ represents the relative abundance of gene *i* in sample *S*, *b*_*i*_ represents the copy number of gene *i* in the sequenced data from sample *S*, *L*_*i*_ represents the length of gene *i*, and *X*_*i*_ represents the times which gene *i* can be identified in sample *S* (the number of mapped contigs).

In our sequence-based profiling analysis, only alignments with followed features could be accepted: (i) an entire of a pair-end read could be mapped onto a gene with the correct insert-size; (ii) one end of the pair-end read could be mapped onto the end of a gene, only if the other end of read was mapped outside the region of gene. Thus, the mapped read was regarded as one copy.

Then, the relative gene abundance of each level from the same taxonomy was added together, and the total relative abundance was regarded as the content of this taxonomy in a certain sample to construct the taxonomy profile of relative abundance on this sample.

#### Criteria for a positive result of shotgun metagenomic sequencing

The results should be considered positive by the following steps. First, at least three copies were mapped to the pathogens whose relative abundance should exceed their own threshold set up by our preliminary data were included into the next. The threshold relative abundance of each pathogen was identified by shotgun metagenomic sequencing in samples of healthy volunteers with the highest abundance in genus level in the preliminary experiments (Table S1). Then, the identified pathogens ranked top five for bacteria, virus, parasite and top ten for fungi in relative abundance were screened out as candidate pathogens for further consideration. After primary analysis, if the candidate microbes were commonly reported in the certain disease of that patient or in accordance with the patient’s clinical manifestations, they would be considered as causative pathogens (with the help of experienced laboratory doctors).

#### Prediction and analysis of ARGs

To identify potential in the genomic sequence of specimens, each specimen’s genomic sequence with relative abundance higher than 1% was screened (Couto et al., 2018) and aligned with the protein sequences of antibiotic resistance genes in the Comprehensive Antibiotic Resistance Database (CARD; https://card.mcmaster.ca) (Jia et al., 2017).

### Statistical analysis

Statistical analyses were performed using SPSS software (version 19.0). For characteristics of patients, continuous data was described by median and interquartile range (*IQR*); categorical data was described by number (*n*) and percentage (%), and compared by *Chi-square* test or *Fisher’s* exact test. A two-tailed *p*-value of < 0.05 was taken as a cut-off for statistical significance.

## Results

### Criteria for evaluation

To compare easily, specimen was cultured (for detection of both bacterial and fungal growth) and extracted DNA (for PCR amplification) at the same time if it met the inclusion criteria. Specimens were selected to perform shotgun metagenomic sequencing if expected bands were seen in the PCR test (>1,000 bp for bacteria, and >750 bp for fungi, respectively). For the purposes of the validation, culture results of PCR-positive specimen were reserved as a standard to compare against shotgun metagenomic sequencing results. Contaminated specimen(s) were identified by the presence of bands in the negative controls or PCR blank, and were excluded from sequencing analysis. Specimens that did not pass the quality control before bioinformatics analysis were considered conflicts and ruled out from the final analysis. Discrepancies might be seen between culture-negative results and shotgun metagenomic sequencing results because of slow growing microorganisms, anaerobic bacteria, or other fastidious organisms.

The results were defined as “matches” when the culture isolates were identified in the shotgun metagenomic sequencing testing. If the culture-positive results were not detected in the shotgun metagenomic sequencing analysis or culture-negative results were detected in the shotgun metagenomic sequencing analysis, they were designated as “conflicts”. The species tested in the shotgun metagenomic sequencing analysis were finally confirmed by a laboratory doctor when combined with clinical manifestations.

### General characteristics

From the 89 specimens of body fluids prospectively included for this study, 25 specimens showed positive in PCR reaction, but five of them were excluded from the sequencing workflow because of insufficient sample size, resulting in a final comparative analysis of 20 specimens (one specimen per patient; see Fig. 2). The demographic features of these patients are listed in Table 1. The sample types included five abscess specimens, five CSF specimens, four bile specimens, two bone marrow specimens, two joint aspirate specimens, one ascites specimen and one pleural fluid specimen. Since Xiangya Hospital is a superior referral hospital, only two patients did not receive empirical antibiotic treatment before sampling.

### Overview of comparative analysis

**Figure 3:**
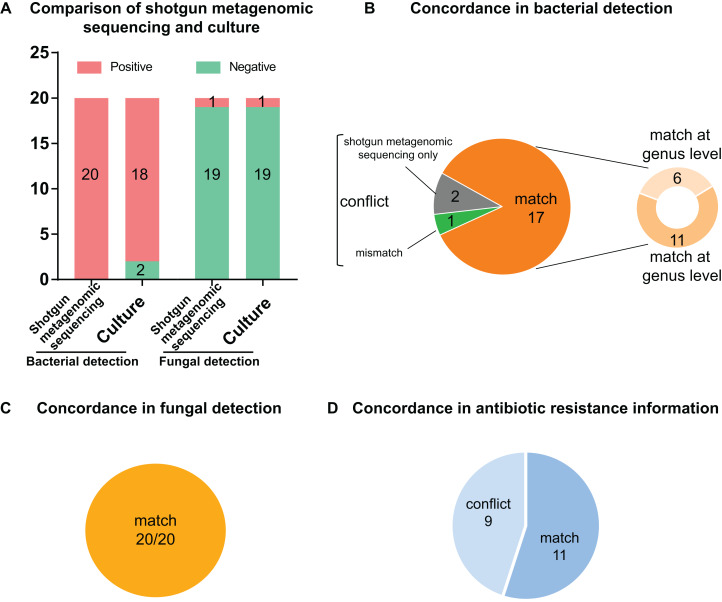
Comparison and concordance analysis between shotgun metagenomic sequencing and culture in pathogen detection and drug resistance information. Comparison and concordance analysis between shotgun metagenomic sequencing and culture in pathogen detection and drug resistance information. (A) The number of positive specimens (y-axis) for pairwise shotgun metagenomic sequencing and culture is plotted against bacterial detection and fungal detection (x-axis) (*n* = 20). (B, C and D) Pie chart demonstrating the positivity distribution of shotgun metagenomic sequencing and culture for all specimens from bacterial detection (B), fungal detection (C), and antibiotic resistance information (D).

Of the 20 PCR-positive specimens, one specimen was reported as no pathogen growth (both bacterial-negative and fungal-negative), one specimen was reported as fungal growth (fungal-positive but bacterial-negative), and 18 specimens were bacterial-positive in conventional culture methods. Meanwhile, all 20 specimens were detected bacterial infection in the shotgun metagenomic sequencing analysis, including one case of bacterial and fungal co-infection. Thus, the match rate between shotgun metagenomic sequencing and culture is 17/20 in bacterial detection, and 20/20 in fungal detection. The comparison of culture and shotgun metagenomic sequencing results is shown in Fig. 3A, and a comprehensive list with culture and sequencing results of each specimen and the relative abundance of each specimen are available in Tables S2 and S3.

### Analysis of culture positive results

Of the 18 bacterial culture positive specimens, 6 specimens at genus level and 11 specimens at species level showed consistent pathogen results between culture and shotgun metagenomic sequencing analysis, making a match rate of 17/18 (see Fig. 3B). In addition, the fungal culture positive specimen was positive for both bacterial and fungal identification by shotgun metagenomic sequencing workflow, with a match of fungal result but conflict of bacterial result. Therefore, the match rates of fungal culture positive results were 1/1 (see Fig. 3C). Specific information of the conflicting results is shown in Table 2. For conciseness, in spite of huge amounts of species identified from metagenomics analysis, only species of the highest abundance and/or clinical relevance are displayed.

**Table 2:** The details of conflicting results between culture and shotgun metagenomic sequencing approach.

Specimen type	Culture results		Shotgun metagenomic sequencing results		
	Bacterial results	Fungal results	Species level of bacterial results	Genus level of bacterial results	Fungal results
Ascites (P5)	(−)	*Candida albicans*	*Pseudomonas aeruginosa* (19.104%)*, *Klebsiella pneumoniae (9.090%*	*Pseudomonas* (39.559%)*	*Candida albicans* (78.152%)
Necrotic tissue (P13)	*Pseudomonas putida*	(−)	*Escherichia coli* (9.627%)*, *Acinetobacter baumannii* (6.225%)	*Acinetobacter* (14.583%)*	(−)
Necrotic tissue (P20)	(−)	(−)	*Acinetobacter baumannii* (7.263%)*	*Acinetobacter* (24.197%)*	(−)

**Notes:**

* Species of highest abundance.

Species listed here are depended on species matching with highest abundance, and most clinical relevance.

### Analysis of culture negative result

The two bacterial culture negative specimens processed by shotgun metagenomic sequencing procedure were reported bacterial growth, which meant it conflicted with culture result (0/2) (see Fig. 3B). All 19 fungal culture negative specimens were not identified fungal growth in the shotgun metagenomic sequencing workflow (19/19) (see Fig. 3C). Details of these sequencing positive, culture negative specimens are revealed in Table 2. Just species of the highest abundance and/or clinical relevance are displayed, although a large number of species are detected in the metagenomics process.

### Comparative analysis of drug resistance information

In order to analyze drug-resistance-related information, we compared ARGs obtained from sequencing data with clinical antimicrobial susceptibility tests. The ARGs of each specimen were aligned and annotated in the CARD database, and the top 10 genes with relative abundance higher than 1% were screened and used for analysis. Since annotated information of genes was antibiotic types and drugs of susceptibility testing were more specific, antibiotic classes were used as the basis for evaluating the consistency of drug resistance.

If more than half of the antibiotic classes of susceptibility tests were the same as the annotated antibiotic classes of ARGs, the results were considered “consistent”; otherwise, the results were considered “conflict”.

As shown in Fig. 3D, 11 specimens showed consistent resistant antibiotic classes between sequencing results and susceptibility testing results, while nine specimens showed conflict between phenotype and genotype. Detailed information on the conflicting drug resistance conditions is shown in Table 3, and a comprehensive list with susceptibility test and sequencing prediction of each specimen is available in Table S4.

**Table 3:** The details of conflicting results between antibiotic resistance phenotypes and genotypes by susceptibility testing and shotgun metagenomic sequencing approach.

No	Resistance information in susceptibility tests		Resistance information in shotgun metagenomic sequencing	
	Susceptibility testing results*	Antibiotic classes of drugs	Resistance gene types from CARD^#^	Antibiotic classes of genes
P3	CZO, NIT	**Cephalosporin**, nitrofuran	*acrB, bl2be_shv2, tetA, sul2, mdtF, tetC, mdtO, mdtE, mdtP, aph(6)-Id*	Tetracycline, penam, **cephalosporin**, glycylcycline, rifamycin, chloramphenicol, fluoroquinolone, sulfonamide, macrolide, aminoglycoside
P4	(−)	(−)	*ermC, sul1, acrB, bacA, mexB, mexW, mexA, qnrB, mexI, mexD*	Macrolide, lincosamide, streptogramin, sulfonamide, diaminopyrimidine, tetracycline, penam, cephalosporin, glycylcycline, rifamycin, chloramphenicol, fluoroquinolone, monobactam, aminoglycoside
P5	(−)	(−)	*mexA, sul1, bl1_pao, acrB, mexB, bacA, mexD, qnrB, mexI, tetD*	Penam, sulfonamide, diaminopyrimidine, tetracycline, cephalosporin, monobactam, chloramphenicol, fluoroquinolone, macrolide, cephamycin, glycylcycline, rifamycin, aminoglycoside
P9	AMP, AMC, NIT	Penam, β-lactamase, nitrofuran	*bacA, adeA, adeB, aph(3′)-Ia, ermC, tet39, lnuA, msrA*	Aminoglycoside, macrolide, lincosamide, streptogramin, tetracycline, glycylcycline
P12	AMK, AMP, AMC, TZP, CRO, FEP, FOX, ATM, IMP, TOB, GEN, CIP, SMZ-TMP, NIT	**Aminoglycoside**, penam, β-lactamase, cephalosporin, monobactam, fluoroquinolone, **sulfonamide**, **diaminopyrimidine**, nitrofuran	*aph(3′)-Ia, aph(6)-Id, tetB, aph33ib, bacA, adeB, adeA, sul2, adeC, sul1*	**Aminoglycoside**, tetracycline, streptogramin, **sulfonamide**, **diaminopyrimidine**, glycylcycline
P17	CSL, SAM, CAZ, MEM, AMK, AMP, TZP, AMC, CRO, FEP, FOX, IMP, GEN, TOB, LVX, CIP, SMZ-TMP, NIT	β-lactamase, cephalosporin, penam, **aminoglycoside**, **aminoglycoside**, fluoroquinolone, **sulfonamide**, **diaminopyrimidine**, nitrofuran	*aph(3′)-Ia, tetB, aph33ib, aph(6)-Id, bacA, sul1, ant(3″)-Ia, adeB, adeC, catB3*	**Aminoglycoside**, tetracycline, streptogramin, **sulfonamide**, **diaminopyrimidine**, glycylcycline, chloramphenicol
P18	CZO, FOX, SMZ-TMP, NIT	Cephalosporin, **sulfonamide**, **diaminopyrimidine**, nitrofuran	*tet39, bacA, aph(6)-Id, aph33ib, sul2*	Tetracycline, aminoglycoside, streptogramin, **sulfonamide**, **diaminopyrimidine**
P19	(−)	(−)	*tet39, lnuA, bacA*	Tetracycline, lincosamide
P20	(−)	(−)	*tet39, lnuA, ermC, blaZ, bacA, sul1, aph(3′)-Ia, ermB, msrA, qacB*	Tetracycline, lincosamide, macrolide, streptogramin, penam, fluoroquinolone, aminoglycoside

**Notes:**

* Only non-susceptibility is listed.

# Resistance genes listed here are the top 10 genes with relative abundance higher than 1%.

Reference number cells were highlighted in grey if resistance information of whole metagenome-shotgun sequencing results conflicted with susceptibility tests, where conflicts were defined by when the consistency of the antibiotic classes in susceptibility tests and annotated by CARD database was less than or equal to 50%. Consistent resistant classes between susceptibility tests and resistance genes were shown in bold. AMC, amoxicillin-clavulanic acid; AMK, amikacin; AMP, ampicillin; ATM, amikacin; CAZ, ceftazidime; CIP, ciprofloxacin; CLI, clindamycin; CRO, ceftriaxone; CSL, cefpoerazone-sulbactam; CTT, cefotetan; CXM, cefuroxime; CZO, cefazolin; ERY, erythromycin; ESBLs, extended spectrum beta-lactamases; ETP, ertapenem; FEP, cefepime; FOX, cefoxitin; GAT, gatifloxacin; GENhl, gentamicin high-level; IMP, imipenem; LVX, levofloxacin; MEM, meropenem; MFX, moxifloxacin; NIT, nitrofurantoin; OXA, oxacillin; PEN, penicillin; PIP, piperacillin; SAM, ampicillin-sulbactam; SMZ-TMP, sulfamethoxazole-trimethoprim; TCY, tetracycline; TOB, tobramycin; TZP, piperacillin-tazobactam.

## Discussion

Accurate and early identification of the pathogen driving infections is vitally important for guiding the diagnosis of disease, the decision of nursing management, and the application of proper antimicrobial therapy. Although culture-based approach regards as “gold standard” of microbes detection currently, it remains to be limited by many factors, which leads to the low positive rate or undetectable result (Abayasekara et al., 2017; Decuypere et al., 2016; Kirn & Weinstein, 2013; Vincent et al., 2015). Moreover, false negative results may often emerge in culturing anaerobic bacteria or *M. Tuberculosis*, which require special culture media or long-time culture (Olaru et al., 2018). Thus, a need of optimizing culture methods and seeking for better approaches is put on the agenda.

Recently, more and more studies were published on the use of shotgun metagenomic sequencing technology for the identification of pathogens in clinical samples (Kohl et al., 2015; Naccache et al., 2014; Wilson et al., 2014), making it as a potential tool to speed pathogen discovery. Several case reports and studies showed potential of shotgun metagenomic sequencing approach to enhance diagnosis of difficult-to-detect pathogens in various conditions. To these aims, this study intends to explore the advantages and disadvantages of shotgun metagenomic sequencing technology compared with the conventional culture method in pathogen identification and resistance information, and expand evidence of its potential application in real-life clinical settings.

In the present study, we comprehensively compared pathogen diagnosis and resistance information by shotgun metagenomic sequencing and culture in a pairwise manner, and found that shotgun metagenomic sequencing approach has advantages in several respects. First, shotgun metagenomic sequencing is known for its rapid and sensitive detection in diagnosing fungi and bacteria, and its short turn-around time might speed up early clinical decision and precise antimicrobial treatment. Then, shotgun metagenomic sequencing could provide ARGs information as well as resistance antibiotic types by bioinformatics pipeline, making it possible to apply proper antimicrobial therapy in time.

Of the 20 specimens in the study, fungi detection showed complete consistent results between these two methods. In terms of bacterial detection, due to the related technical complexity in taxonomic classification, both bacterial identification at genes level and species level is of great value for comparison. We found that shotgun metagenomic sequencing identified extra pathogens in two specimens when conventional culture failed to get positive result, and reported 17 relatively consistent results in 18 bacterial positive culture results (including 11/18 at species level and 6/18 at genus level). Due to the preliminary selection of PCR-positive specimens, shotgun metagenomic sequencing had a high negative predictive value than conventional culture, as is similar to previous studies (Parize et al., 2017; Thoendel et al., 2018; Zhang et al., 2020).

Except for those consistent results, three specimens (P5, P13, and P20) showed completely conflict between shotgun metagenomic sequencing and conventional culture. Besides, six specimens (P1, P3, P6, P10, P18, and P19) only matched at genus level, showing a widely rather than narrow consistence. Of note, unlike culture, more than one strain was identified in a specimen in the most of shotgun metagenomic sequencing workflow. The metagenomics results of culture negative specimens were further evaluated by the clinical microbiologist, which was finally concluded that the negative results of culture might be due to the application of antibiotics before admission and higher sensitivity of shotgun metagenomic sequencing workflow. Similar finding were also confirmed by previous studies, e.g., Miao et al. (2018) and Zhang et al. (2019) revealed shotgun metagenomic sequencing detected more potential microbes than culture in CNS infection cases with empirical antibiotic treatment.

Furthermore, the inexistent organisms in the culture identified by shotgun metagenomic sequencing pipeline may be attributed to growth inhibition induced by antibiotics usage, growth restriction by inappropriate culture conditions, or microbial interaction (Abayasekara et al., 2017; Decuypere et al., 2016). Our results indicated that the shotgun metagenomic sequencing technology was able to detect pathogens that could not be directly identified by conventional culture, either for technical or practical reasons. That is to say, in some cases shotgun metagenomic sequencing may be more sensitive than conventional culture in identifying pathogens. Consequently, it should be realized that culture-negative specimens do not actually mean pathogen-free, but it might reflect the low sensitivity of culture-based methods or non-culturable characteristics of some specimens (e.g., prior antibiotics treatments). So, one of the applications of shotgun metagenomic sequencing may be to detect the specimens remaining negative in standard culture either for technical reasons, or for unsuspected organisms so that it can be used as a potential diagnostic tool with an added value.

Since we insisted on aseptic operation in our pipeline from beginning to end (Graf et al., 2016; Street et al., 2017), we could firmly believe the results of shotgun metagenomic sequencing were accurate and true. Based on the above premise, we found out that most of the results by shotgun metagenomic sequencing were multiple bacterial infections, and the culture results were usually one single strain, which was also described by other studies (Yan et al., 2020; Yin et al., 2017; Zhang et al., 2020). Emerging evidence also exists that multiple microbial co-infections often occurred in patients with severe infections (Herberg et al., 2016; Song, Xu & Shen, 2016; Yin et al., 2017), and bacterial toxins could cause serious consequences even in the absence of live bacteria (de Punder & Pruimboom, 2015). So, here come the questions. Do all these bacteria detected from shotgun metagenomic sequencing exist in specimens or infected sites? Are they all the sources of infection? Among these conflict results, are the culture results or shotgun metagenomic sequencing results more credible? However, because the existing technical approaches cannot explain or verify these issues, we cannot blindly hold the idea that one of them is more credible to be believed, but can only judge them in combination with clinical manifestations.

Miao et al. (2018) recently performed a similar study using shotgun metagenomic sequencing retrospectively to diagnose infectious disease for all suspected patients. However, due to their wider inclusion of all patients with suspected infections, they revealed that shotgun metagenomic sequencing approach did not identify as much more positive results as culture could, suggesting that shotgun metagenomic sequencing for common bacterial detection might not be as advantageous as it for viral and fungal might, and it is recommended as a supplement to conventional culture rather than replacement.

In addition, resistance information of causative pathogen is an important part of microbiological diagnosis, however, fewer studies except for several case reports investigated the performance of it by shotgun metagenomic sequencing in infectious diseases. Thus, we compared the drug resistance state in each sample in parallel between sequencing test and susceptibility test, to evaluate the application value of this novel technology in clinical practice ulteriorly. In order to enhance comparability in the present study, antibiotic classes were used as the basis for evaluating the consistency of drug resistance between shotgun metagenomic sequencing and drug susceptibility test. We confirmed more than half specimens had resembling resistant drug types, especially in those concordant specimens. The conflicting results (P3, P4, P5, P9, P12, P17, P18, P19, and P20) between phenotypes (susceptibility tests) and genotypes (annotated ARGs) are probably results from the complexity of drug resistance mechanisms. For example, the mechanism of bacterial resistance is not only caused by gene mutation, also may be changes in point mutations, gene expression changes, and posttranslational modifications, etc. (Grumaz et al., 2016); the applicability of the CARD database for detecting ARGs is only for genes per se resistant to antibiotics. So the detection of ARGs cannot fully represent the actual drug resistance of microbes. Notably, a shortcoming of resistant genes’ analysis is that they do not provide an accurate measure of antibiotics susceptibility; in particular, if the presence of ARGs or mutations confer inducible resistance, phenotypic testing will still be required (Jenkins & Schuetz, 2012; Lefterova et al., 2015). Meanwhile, a resistance gene phenotype cannot be predicted in case that it has not been characterized genetically or is not in an existing database. Therefore, shotgun metagenomic sequencing-based ARGs’ detection seems to be feasible, but it is unlikely to replace antimicrobial susceptibility tests at present.

Overall, these two approaches have their own strengths and weakness in the process of clinical application. For the moment, the combination of these two approaches with clinical evidences is the “best practice” for the diagnosis of pathogenic microorganisms, especially for those infectious diseases or critical illness that are difficult to diagnose.

There are still several limitations to this study. Although costs per base are steadily dropping, sequencing-based approaches are still comparatively expensive. Therefore, a small size of samples in our study was enrolled to achieve a relatively reliable sequencing depth for the detection of causative microbes (Thoendel et al., 2018). Then, a variety of specimen types was contained. Considering bias could have happened if only one type of specimen had been used, it was profitable to use different types of specimens to obtain unbiased analysis. Moreover, blood specimens were not contained in this study. Owing to the low positive rate of blood culture and large amount of host cells in samples, blood samples were not included in this study. Thus, the standards in our study should be thoroughly modified and validated in the expanded studies before applying in clinical practice.

## Conclusions

In conclusion, this study suggested the specificity of shotgun metagenomic sequencing for the identification of causative pathogens, and provided new information and experimental evidence for the application of shotgun metagenomic sequencing approach in pathogenic identification of infectious diseases, especially for the cases where standard culture approaches failed to identify microorganisms.

## Supplemental Information

10.7717/peerj.11699/supp-1 Supplemental Information 1 The threshold of taxonomy relative abundance in genus level.

10.7717/peerj.11699/supp-2 Supplemental Information 2 The details of specimen type in comparative analysis of culture and shotgun metagenomic sequencing results.

10.7717/peerj.11699/supp-3 Supplemental Information 3 Taxonomic relative abundance of all specimens by shotgun metagenomic sequencing.

10.7717/peerj.11699/supp-4 Supplemental Information 4 Antibiotic resistance phenotypes and genotypes detected by susceptibility testing and shotgun metagenomic sequencing approach.

## Additional Information and Declarations

### DNA Deposition

The following information was supplied regarding the deposition of DNA sequences: The primers of 16S rDNA, ITS1 and ITS4 were purchased from Sangon Biotech, Shanghai, China.

### Data Availability

The following information was supplied regarding data availability: Data are available in the National Center for Biotechnology Information (NCBI) repository: PRJNA527188.
